# The preventive effect of antiplatelet therapy in acute respiratory distress syndrome: a meta-analysis

**DOI:** 10.1186/s13054-018-1988-y

**Published:** 2018-03-08

**Authors:** Yingqin Wang, Ming Zhong, Zhichao Wang, Jieqiong Song, Wei Wu, Duming Zhu

**Affiliations:** 1 Department of Critical Care Medicine, Zhongshan Hospital, Fudan University, 180 Fenglin Road, Shanghai, China; 2 Liver Cancer Institute, Zhongshan Hospital, Fudan University, Shanghai, China

**Keywords:** Acute respiratory distress syndrome, Antiplatelet drugs, Prevention, Meta-analysis

## Abstract

**Background:**

Acute respiratory distress syndrome (ARDS) is a life-threatening condition with high mortality that imposes a serious medical burden. Antiplatelet therapy is a potential strategy for preventing ARDS in patients with a high risk of developing this condition. A meta-analysis was performed to investigate whether antiplatelet therapy could reduce the incidence of newly developed ARDS and its associated mortality in high-risk patients.

**Methods:**

The Cochrane Central Register of Controlled Trials (CENTRAL), PubMed, Embase, Medline, and the Web of Science were searched for published studies from inception to 26 October 2017. We included randomized clinical trials, cohort studies and case-control studies investigating antiplatelet therapy in adult patients presenting to the hospital or ICU with a high risk for ARDS. Baseline patient characteristics, interventions, controls and outcomes were extracted. Our primary outcome was the incidence of newly developed ARDS in high-risk patients. Secondary outcomes were hospital and ICU mortality. A random-effects or fixed-effects model was used for quantitative synthesis.

**Results:**

We identified nine eligible studies including 7660 high-risk patients who received antiplatelet therapy. Based on seven observational studies, antiplatelet therapy was associated with a decreased incidence of ARDS (odds ratio (OR) 0.68, 95% confidence interval (CI) 0.52–0.88; *I*^2^ = 68.4%, *p* = 0.004). In two randomized studies, no significant difference was found in newly developed ARDS between the antiplatelet groups and placebo groups (OR 1.32, 95% CI 0.72–2.42; *I*^*2*^ = 0.0%, *p* = 0.329). Antiplatelet therapy did not reduce hospital mortality in randomized studies (OR 1.15, 95% CI 0.58–2.27; *I*^*2*^ = 0.0%; *p* = 0.440) or observational studies (OR 0.80, 95% CI 0.62–1.03; *I*^2^ = 31.9%, *p* = 0.221).

**Conclusions:**

Antiplatelet therapy did not significantly decrease hospital mortality in high-risk patients. However, whether antiplatelet therapy is associated with a decreased incidence of ARDS in patients at a high risk of developing the condition remains unclear.

**Electronic supplementary material:**

The online version of this article (10.1186/s13054-018-1988-y) contains supplementary material, which is available to authorized users.

## Background

Acute respiratory distress syndrome (ARDS) is a life-threatening condition with an approximate 40% hospital mortality rate [1], costing 3.6 million hospital days annually in the USA and accounting for 10.4% of intensive care unit (ICU) admissions around the world [1, 2]. In recent decades, progress has been achieved in the development of possible treatments for ARDS, including protective ventilation strategies, prone positioning, neuromuscular blockade, and extracorporeal membrane oxygenation [1]. Some pharmacologic compounds have also been suggested to be effective in ARDS prevention [3]. However, no Food and Drug Administration approved treatment for ARDS is currently available [4].

ARDS is characterized by uncontrolled inflammation and coagulation with increased capillary permeability, inflammatory cell accumulation in the lung compartments, and pulmonary microvascular coagulopathy [5]. Platelets play a key role in the pathogenesis and resolution of ARDS as mediators of hemostasis and coagulation, modulators of inflammation and the immune system, and defenders of microbes [6–8]. Antiplatelet therapy can attenuate lung injury by impeding platelet activation and surface adhesion protein expression, such as glycoprotein IIb/IIIa receptor, P-selectin, and intracellular adhesion molecule 1 (ICAM-1) [9], which is a key step in microvascular thrombus formation and tissue injury [10, 11]. Platelet inhibition also suppresses the secretion of inflammation mediators such as cytokines, chemokines and granules [11, 12] and thereby attenuates inflammation in the lung and its interaction with the immune system. Moreover, platelet activation is reported to initiate the innate immune response through pathogen recognition patterns and proinflammatory neutrophil extracellular trap (NET) formation in acute lung injury [13]. In addition, platelets can produce functional progeny through self-regulation [14] and generate a positive feedback loop, amplifying both the homeostatic and inflammatory responses [15]. Inactivation of platelets may impede this positive feedback and improve outcomes among high-risk patients.

Preclinical studies have identified beneficial effects of antiplatelet therapy in ARDS prevention, as evidenced by improved oxygenation, diminished lung edema, attenuated inflammation, and increased survival [5]. In contrast, antiplatelet therapy also exhibited biphasic behavior in an animal study, with benefit in the early phase followed by worsening of gas exchange in the late phase [16]. Pretreatment with antiplatelet therapy has also been reported to increase inflammation in the lung [17]. The results of a meta-analysis based on observational studies suggested that antiplatelet therapy with aspirin was significantly associated with reduced incidence of ARDS in mixed critically ill patients [5]. However, a recent, well-designed, randomized controlled clinical trial showed that neither the incidence of ARDS at 7 days nor the incidence of adverse effects was significantly different between antiplatelet therapy and placebo groups [18]. Therefore, the effect of antiplatelet therapy on ARDS prevention remains controversial. The objective of the present meta-analysis was to compare the incidence of newly developed ARDS and mortality between patients with and without antiplatelet therapy, who were at a high risk of ARDS.

## Methods

### Protocol and search strategy

This meta-analysis was conducted and reported in accordance with Preferred Reporting Items for Systematic Reviews and Meta-Analyses (PRISMA) [19] (see Additional file 1). A study protocol was established prior to the literature search (see Additional file 2).

Electronic databases including the Cochrane Central Register of Controlled Trials (CENTRAL), PubMed, Embase, Medline, and the Web of Science were searched for published studies. The last search was performed on 26 October 2017 (details of the search strategies are provided in Additional file 2). The reference lists of review articles were manually screened for other potential studies.

### Study selection and data extraction

Randomized and observational studies (prospective or retrospective cohort studies and case-control studies) were included. The inclusion criteria were adult patients presenting to the hospital or ICU with a high risk of ARDS, administration of antiplatelet therapy at any time or dose, comparison between patients with and without antiplatelet therapy, and a report of newly developed ARDS. High-risk factors for ARDS identified in previous studies to be closely associated with the development of ARDS were defined as sepsis, non-cardiogenic shock, trauma, high-risk surgery, aspiration, pneumonia, pancreatitis, and massive transfusion [1, 20–22]. The exclusion criteria were inclusion of patients without a risk factor for ARDS, inclusion of patients who had already developed ARDS upon arrival to the hospital or ICU, lack of a comparison group, and healthy volunteer studies. Non-English-language studies were also excluded. The primary outcome was the incidence of newly developed ARDS in high-risk patients. Secondary outcomes were hospital and ICU mortality.

Two authors (WW and JS) independently screened the titles and abstracts of records to identify potential studies. Baseline patient characteristics, interventions, controls, and outcomes were extracted after full-text review using a standard data extraction form (see Additional file 2). Disagreements were resolved by discussion with a third author (ZW).

### Quality assessment

The risk of bias in randomized studies was evaluated with the Cochrane Collaboration tool for assessing the risk of bias [23]. For observational studies, the Newcastle-Ottawa Scale (NOS) for cohort and case-control studies was applied accordingly [24]. Observational studies with NOS scores of 8 or 9, 6 or 7, and < 6 were judged as low, medium, and high risk of bias, respectively [25]. Grading of Recommendations Assessment, Development, and Evaluation (GRADE) was used to judge the quality of evidence for each outcome [26]. The quality of evidence was judged as high, moderate, low, or very low using GRADE profiler 3.6 (GRADEpro; McMaster University 2014, Hamilton, Canada).

### Data analysis

Randomized and observational studies were analyzed separately. If adjusted odds ratios (ORs) were reported, then they were extracted and used for data combination. Otherwise, calculated ORs were used for data combination. The chi-square test was used to combine data with 95% confidence intervals (CIs). A fixed-effect model was used for combination when heterogeneity was absent according to the *Q* statistic (*p* ≥ 0.1) and Higgins *I*^2^ test (*I*^*2*^ < 30%). Otherwise, a random-effect model was used. Pre-specified subgroup analyses were conducted for the primary outcome. Sensitivity analysis was performed on the primary outcome by omitting one study at a time to assess the robustness of the results [27, 28]. A funnel plot was not applicable because of the limited number of studies included in this analysis [29]. Publication bias was examined by Egger’s test. A *p* value < 0.05 was considered statistically significant. All statistical analyses were performed in STATA, version 13.0 (Stata Corporation, College Station, TX, USA).

## Results

**Fig. 1:**
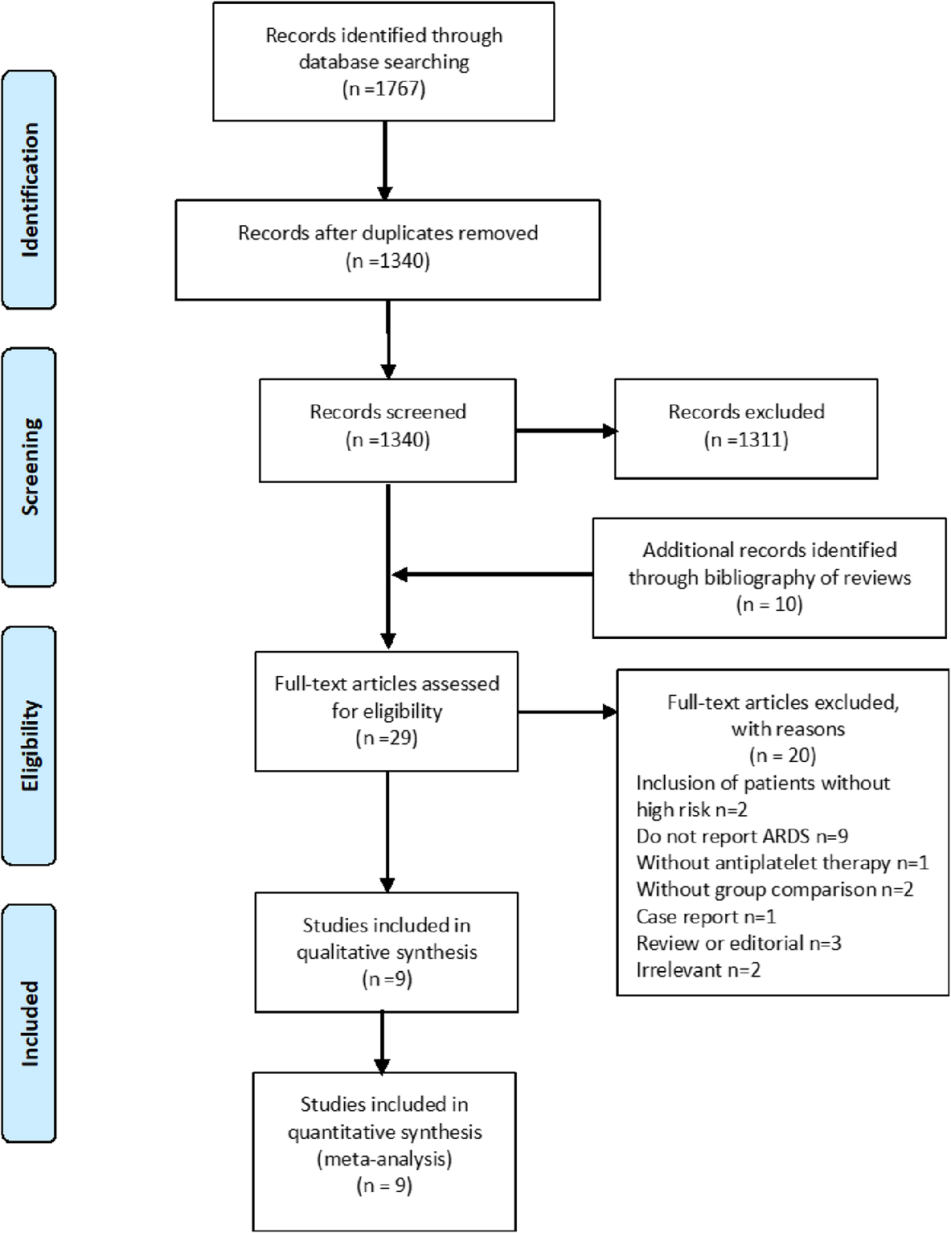
Search, inclusion and exclusion flow diagram. ARDS, acute respiratory distress syndrome

Our search identified 1767 relevant publications. After duplicates were removed, 1340 study titles and abstracts were screened, and 29 studies were selected for full-text review. Finally, we included 9 studies (2 randomized studies [18, 30] and 7 observational studies [11, 31–36]) with 7660 patients in this meta-analysis. Details of the study selection procedure are shown in Fig. 1 (see studies excluded and reasons for exclusion in Additional file 3: Table S1). Study characteristics are shown in Table 1.

**Table 1:** Main characteristics of the randomized and observational studies

Author, year	Study design	Number of centers; country	Study scale, total number (anti/non-anti)	Study population	Definition of antiplatelet therapy	Definition of ARDS
Kor, 2016 [18]	RCT	16; USA	390 (195/195)	Patients aged 18 years or older with an elevated risk of developing ARDS (LIPS ≥4)	Aspirin	Berlin definition
Vincent, 1985 [30]	RCT	1; Belgium	43 (16/17)	Patients developed an episode of non-cardiogenic shock due to hemorrhage, trauma or sepsis	Dipyridamole	Severe hypoxemia and generalized lung infiltrates with PAWP <14 mmHg
Mazzeffi, 2015 [32]	Retrospective cohort	1; USA	375 (181/194)	Patients received aortic valve replacement surgery with cardiopulmonary bypass	Aspirin	Berlin definition
Chen, 2015 [31]	Prospective cohort	1; USA	1149 (287/862)	Patients admitted to the ICU with a high risk of ARDS	Aspirin	AECC definition
Valerio, 2013 [11]	Retrospective cohort	1; USA	651 (272/379)	Patients admitted to the medical ICU with severe sepsis and septic shock on antiplatelet therapy before diagnosis of severe sepsis or septic shock.	Aspirin, clopidogrel	AECC definition
Kor, 2011 [33]	Prospective cohort	20; USA, 2; Turkey	3855 (976/2879)	Adult, non-surgical patients admitted to the hospital with at least one major risk factor for ALI	Aspirin	AECC definition
Erlich, 2011 [34]	Retrospective cohort	1; USA	161 (79/82)	Adult patients admitted to the ICU with at least one major risk factor for ALI	Aspirin, clopidogrel bisulfate, ticlopidine, hydrochloride, cilostazol, dipyramidole, anagrelide, or persantine	AECC definition
Ahmed, 2014 [36]	Population-based, nested case-control	2; USA	828 (414/414)^a^	Patients with hospital-acquired ARDS and matched controls	Aspirin, abciximab, cilostazol, clopidogrel,dipyridamole, eptifibatide, ticlopidine	AECC definition
Tuinman, 2012 [35]	Nested case-control	1; Netherlands	218 (109/109)^a^	Adult patients admitted to the medical-surgical ICU diagnosed with TRALI and matched controls	Aspirin, clopidogrel	The 2004 consensus definition of TRALI

*Abbreviations: RCT* randomized controlled trial, *anti/non-anti* antiplatelet/non-antiplatelet, *ICU* intensive care unit, *ARDS* acute respiratory distress syndrome, *ALI* acute lung injury, *LIPS* Lung Injury Prediction Score, *AECC* American-European Consensus Conference, *PAWP* pulmonary artery wedge pressure, *TRALI* transfusion-related acute lung injury

^a^In case-control studies, the numbers represent the total number of patients (number of cases/number of controls)

### Randomized studies

Two randomized studies with 433 patients were identified. In these studies, 39 (9.0%) patients developed ARDS during the study periods. Baseline patient characteristics, antiplatelet interventions and outcomes are shown in Additional file 3: Tables S2–S4. The overall risk of bias was considered unclear as assessed by the Cochrane risk of bias tool (Additional file 4: Figures S1 and S2).

**Fig. 2:**
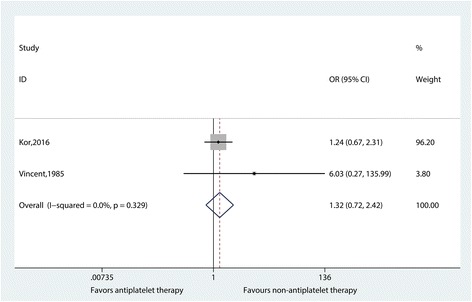
Effect of antiplatelet therapy on newly developed acute respiratory distress syndrome, based on randomized studies. An odds ratio (OR) <1 favors antiplatelet therapy

The incidence of newly developed ARDS (OR 1.32, 95% CI 0.72–2.42; *I*^*2*^ = 0.0%, *p* = 0.329) (Fig. 2) and hospital mortality (OR 1.15; 95% CI 0.58–2.27; *I*^*2*^ = 0.0%; *p* = 0.440) (Additional file 4: Figure S3) was not significantly reduced in the antiplatelet groups, with low heterogeneity among the results. ICU mortality was not pooled due to a lack of data. Subgroup analyses and sensitivity analysis were not performed because only two studies were included.

The evidence was considered low quality for both newly developed ARDS and hospital mortality according to the GRADE system (Additional file 3: Table S5).

### Observational studies

Seven observational studies with a total of 7227 patients were identified. Among them, 844 patients (11.6%) developed ARDS. Patient characteristics, antiplatelet interventions, and outcomes are shown in Additional file 3: Tables S6–S8. The average NOS scores of cohort studies and case-control studies were 7 (6–8) and 7, respectively, and the overall risk of bias was determined to be medium (Additional file 3: Table S9).

**Fig. 3:**
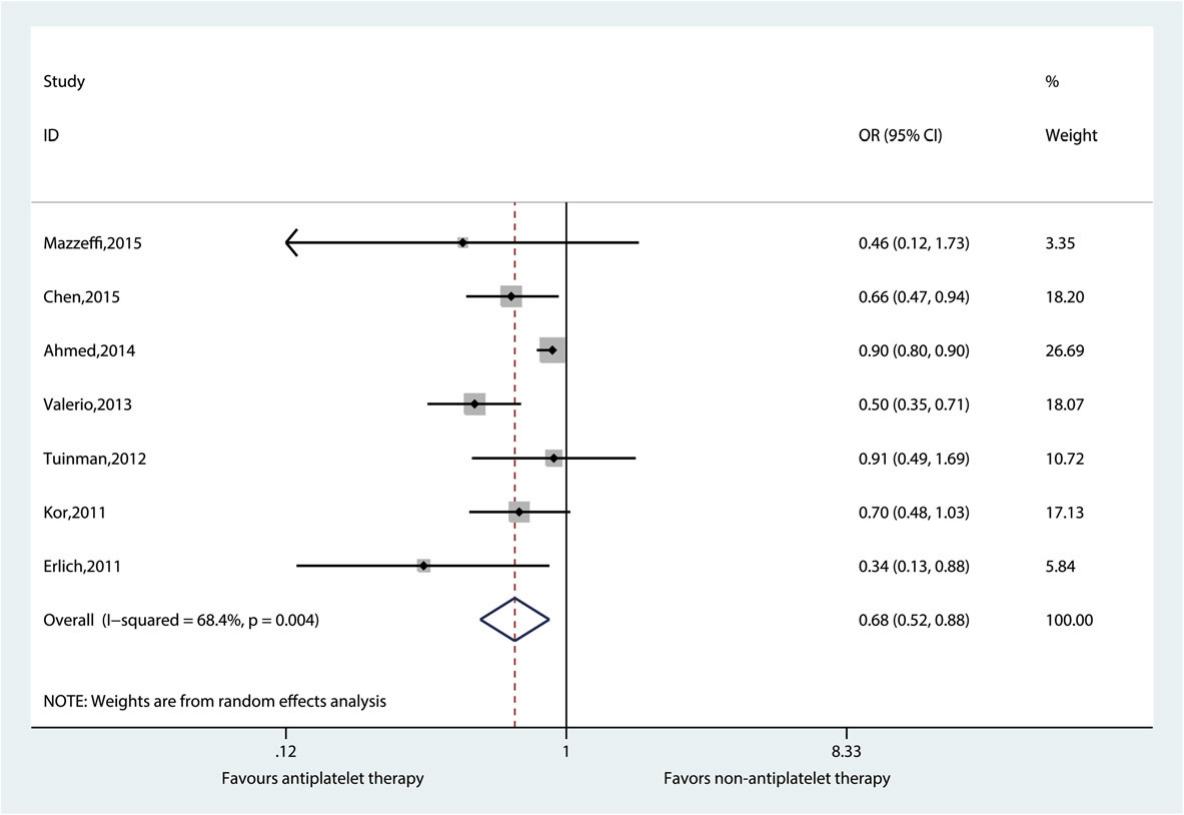
Effect of antiplatelet therapy on newly developed acute respiratory distress syndrome based on observational studies. An odds ratio (OR) < 1 favors antiplatelet therapy

The pooled OR in a random model (OR 0.68, 95% CI 0.52–0.88; *I*^2^ = 68.4%, *p* = 0.004) indicated an association between antiplatelet therapy and a reduced incidence of newly developed ARDS (Fig. 3), with high heterogeneity among the results. Neither hospital mortality (OR 0.80, 95% CI 0.62–1.03; *I*^2^ = 31.9%, *p* = 0.221) nor ICU mortality (OR 0.84, 95% CI 0.63–1.11; *I*^2^ = 0.0%, *p* = 0.851) (Additional file 4: Figures S4–S5) was reduced in antiplatelet groups.

Subgroup analyses demonstrated that heterogeneity was not significant between groups with respect to the definition of antiplatelet therapy, the timing of antiplatelet therapy, the definition of ARDS, or the sample size of the study population. Heterogeneity was not observed in terms of the risk factors for ARDS, except for high-risk surgery. The heterogeneity between studies significantly decreased in the subgroups of study design (*I*^2^ from 68.4 to 0.0%) and high-risk surgery (*I*^2^ from 68.4 to 0.0% and 8.2%). The dose of antiplatelet therapy was not well-documented in the studies. Notably, antiplatelet therapy showed slightly greater protective effects in patients when the analysis was restricted to studies that included patients who used aspirin only, patients receiving antiplatelet therapy before hospitalization, patients with a diagnosis of ARDS according to the American-European Consensus Conference (AECC) definition, and patients without sepsis, shock, pneumonia, aspiration, trauma, high-risk surgery, pancreatitis, or massive transfusion. All results of the subgroup analyses are summarized in Table 2 (forest plots are shown in Additional file 4: Figures S6–S17). Combined ORs were not substantially altered in the sensitivity analysis (Additional file 4: Figure S18). Notably, the heterogeneity between groups significantly decreased (I^2^ = 2.2%) when the study conducted by Ahmed et al. was removed (forest plot shown in Additional Figure S19) [see Additional file 4]. The publication bias was not evident (Egger test *P* = 0.206).

**Table 2:** Summary of the subgroup analyses in observational studies

Subgroups	Number of studies	Sample size	OR	95% CI	P	I^2^
Definition of antiplatelet therapy						
Aspirin only	3	5379	0.67	(0.52–0.86)	0.829	0.0%
Aspirin in combination	4	1848	0.67	(0.44–1.02)	0.003	78.8%
Time of antiplatelet therapy						
Before hospitalization	3	5379	0.67	(0.52–0.86)	0.829	0.0%
Before and/or after hospitalization	4	1848	0.67	(0.44–1.02)	0.003	78.8%
Risk factors for ARDS						
Sepsis						
Yes	5	5703	0.69	(0.50–0.95)	0.004	74.3%
No	2	1524	0.64	(0.46–0.90)	0.603	0.0%
Shock						
Yes	3	4834	0.74	(0.52–0.88)	0.062	63.9%
No	4	2393	0.61	(0.48–0.77)	0.364	5.8%
Pneumonia						
Yes	3	4834	0.74	(0.52–0.88)	0.062	63.9%
No	4	2393	0.61	(0.48–0.77)	0.364	5.8%
Aspiration						
Yes	3	4834	0.74	(0.52–0.88)	0.062	63.9%
No	4	2393	0.61	(0.48–0.77)	0.364	5.8%
Trauma						
Yes	3	4834	0.74	(0.52–0.88)	0.062	63.9%
No	4	2393	0.61	(0.48–0.77)	0.364	5.8%
High-risk surgery						
Yes	3	1411	0.90	(0.85–0.95)	0.609	0.0%
No	4	5816	0.59	(0.48–0.73)	0.352	8.2%
Pancreatitis						
Yes	3	4834	0.74	(0.52–0.88)	0.062	63.9%
No	4	2393	0.61	(0.48–0.77)	0.364	5.8%
Massive transfusion						
Yes	1	218	0.91	(0.49–1.69)	–	–
No	5	7009	0.65	(0.48–0.87)	0.002	73.6%
Definition of ARDS						
Berlin definition	1	375	0.46	(0.12–1.74)	–	–
AECC definition	5	6634	0.66	(0.48–0.89)	0.001	77.9%
The 2004 consensus definition of TRALI	1	218	0.91	(0.49–1.69)	–	–
Size of the population						
Small	5	2223	0.65	(0.44–0.98)	0.004	73.5%
Large	2	5004	0.68	(0.52–0.88)	0.819	0.0%
Study design						
Prospective cohort	2	2502	0.68	(0.52–0.88)	0.819	0.0%
Retrospective cohort	3	1187	0.48	(0.35–0.66)	0.758	0.0%
Case–control study	2	1036	0.90	(0.85–0.95)	0.972	0.0%

*ARDS* acute respiratory distress syndrome, *AECC* American-European Consensus Conference, *TRALI* transfusion-related acute lung injury

The quality of evidence for both newly developed ARDS and hospital mortality was considered low according to the GRADE system, and the quality of evidence for ICU mortality was judged as very low in the cohort studies (Additional file 3: Table S10). In the case-control studies, the quality of evidence for newly developed ARDS was low (Additional file 3: Table S11).

## Discussion

Antiplatelet therapy has been suggested as an option for ARDS prevention, but its impact on patients at a high risk of ARDS remains controversial. We performed a meta-analysis of both randomized and observational studies, focusing on the potential preventive effects of antiplatelet therapy in patients at high risk of ARDS. The analysis of the observational studies suggested that antiplatelet therapy was associated with a reduced incidence of newly developed ARDS. However, the analysis of the randomized studies showed no difference between groups. Antiplatelet therapy was not significantly associated with improved mortality in randomized or observational studies.

The result of the pooled analysis of the randomized studies contradicted the conclusions of the observational studies on the incidence of newly developed ARDS. As randomized studies have a higher evidence level, this finding may suggest that antiplatelet therapy could not protect high-risk patients from ARDS. However, only two randomized studies were included, and the pooled results were mainly influenced by the study conducted by Kor et al. There are some noticeable differences between randomized and observational studies.

All observational studies recruited patients who had received antiplatelet therapy before being admitted to the hospital or the ICU. Although a propensity-adjusted analysis was performed for each observational study, potential heterogeneity among baseline characteristics in the studies cannot be ruled out. Patients on pre-hospital antiplatelet therapy were older and had more comorbidities [11, 31, 33, 34]. Antiplatelet therapy is widely used for secondary prevention of malignant cardiovascular events in older patients with atherosclerotic vascular disease. Atherosclerosis, which is regarded as a chronic low-grade systemic inflammation, may predispose patients to excessive acute inflammation and may increase resistance to inflammation-associated cytokine production and organ failure [37]. A history of vascular disease had an additional benefit on hospital outcomes [37]. In the well-designed randomized studies conducted by Kor et al., potential cofounding effects of pre-hospital use of antiplatelet therapy were mitigated by excluding patients who received antiplatelet therapy at the time of hospitalization [18]. Additionally, the incidence of ARDS in patients who discontinued antiplatelet therapy during hospitalization was not significantly different from that in patients who continued antiplatelet therapy [31]. These findings implied that long-term pre-hospital antiplatelet therapy may protect high-risk patients from ARDS, but this benefit was not evident when patients received antiplatelet therapy after the onset of risk factors. Pre-hospital antiplatelet therapy may protect patients by suppressing platelet aggregation before initial insults occur, but the protective effect may be compromised if this process has already been triggered.

Use of a low dose of 81 mg of aspirin in randomized studies did not significantly reduce the incidence of ARDS [18]. Four of seven observational studies included patients with antiplatelet therapy other than aspirin. A subgroup analysis of the observational studies indicated that patients who used aspirin only had a lower risk of developing ARDS compared with patients using mixed medications. Most patients in the observational studies used low-dose aspirin (81-100 mg), but the data on dosage were insufficient for subgroup analysis. It is reasonable to assume that aspirin will be a prospective treatment. Previous studies have proven that low-dose aspirin is more potent in inhibiting cyclooxygenase I (COX I) than cyclooxygenase II (COX II) [38, 39]; the former is responsible for normal homeostatic processes and the latter inhibits inflammation. Although low-dose aspirin has also been shown to have anti-inflammatory effects due to aspirin-induced lipoxin formation, high-dose aspirin may be more effective in preventing ARDS as all preclinical studies showed beneficial effects of high-dose aspirin in ARDS [5]. Since high-dose aspirin may be related to an increased risk of bleeding, it should be considered with caution pending further clinical investigation.

In another randomized pilot study performed by Vincent et al., patients with circulatory shock received aspirin plus dipyridamole versus aspirin plus placebo [30]. The active comparator was dipyridamole and was therefore included in our analysis. As a small pilot study, they focused on a group of patients with circulatory shock, which is a common risk factor for ARDS in critically ill patients. This study included a relatively large number of patients presenting with hemorrhagic shock, but arterial hypotension was corrected in less than 12 h to avoid coagulation abnormalities in severe or prolonged states of shock. Considering that including patients with hemorrhagic shock and trauma may be confounding, we performed a subgroup analysis on studies with and without patients with shock or trauma. This analysis revealed a slightly greater effect on the decreased incidence of ARDS compared with the overall analysis (0.61 versus 0.68); however, the on pooled analysis of the studies of patients with shock or trauma, the decreased incidence of ARDS was no longer significant (*p* = 0.062). Continuing antiplatelet therapy in patients without hemorrhage may be safe, but antiplatelet therapy in patients with a risk of hemorrhage may be risky.

The present analysis extends the findings of a recent meta-analysis conducted by Panka et al. in which similar inclusion and exclusion criteria were applied [5]. In observational studies, antiplatelet therapy was associated with a reduced incidence of ARDS but not of mortality. A previous meta-analysis conducted by Wang et al. and Mohananey et al. revealed decreased mortality among critically ill patients receiving antiplatelet therapy [25, 40]. Notably, they included studies that did not report the incidence of ARDS [37, 41–51] and possibly included patients at a lower risk of ARDS, which may confound the result [43, 48–51]. Most would expect that if patients were less likely to develop ARDS, then their risk of death would decrease. These results prompted us to review the reported adverse events in the included studies. The most concerning bleeding-related adverse events and acute kidney injury were not significantly different between patients who received or did not receive antiplatelet therapy [11, 18]. The case-control study conducted by Ahmed et al. indicated that adverse events were strongly associated with ARDS development, as were inadequate antimicrobial therapy, mechanical ventilation with injurious tidal volumes, hospital-acquired aspiration, and the volumes of blood products transfused and fluids administered [36]. Vincent et al. also reported that total blood transfusion volume and the ratio of thrombocytopenia was significantly higher in patients who developed ARDS [30]. Most observational studies did not consider these important factors in their analyses [31, 32, 34], potentially contributing to an overestimated benefit of antiplatelet therapy in ARDS prevention.

To our knowledge, this is the first study to explore the potential preventive effects of antiplatelet therapy in patients at a high risk of ARDS in both randomized and observational studies. Nonetheless, this investigation also has important limitations. First, the studies included in this meta-analysis varied considerably in the definition of ARDS, baseline patient characteristics, interventions, and study design. To address this limitation, subgroup analyses and a sensitivity analysis were performed. The subgroup analyses demonstrated that heterogeneity was mainly caused by study design and the inclusion of high-risk surgical patients, but the results remained consistent between subgroups. The sensitivity analysis indicated that the heterogeneity was significantly influenced by the study conducted by Ahmed et al., but the effect of the pooled result did not substantially change after this study was removed. Second, although the search was rigorous and comprehensive and focused on high-risk patients, interpretation of the synthesized results was limited since only two randomized studies and seven observational studies were included. Third, despite a strict selection process, the overall risk of bias was judged as unclear in the randomized studies and as medium in the observational studies. Observational studies have limitations by nature. Therefore, we used the GRADE system to assess the quality of evidence for outcomes. The quality of the evidence provided by the cohort and case-control studies was equal to the quality of the randomized studies, suggesting that the results from the observational studies should also be seriously considered. Furthermore, most of the patients included in this meta-analysis used aspirin alone, and conclusions on other antiplatelet agents should therefore be interpreted with caution. Finally, unpublished studies or conference abstracts were not included, which may be of great significance as well.

## Conclusions

In this analysis, antiplatelet therapy did not significantly reduce mortality in high-risk ARDS patients. However, whether antiplatelet therapy is associated with a decreased incidence of ARDS in patients at high risk of ARDS is still unclear. Therefore, series of large, well-designed randomized trials, especially those focusing on the timing of antiplatelet therapy, the dose of antiplatelet drugs, and the indication for antiplatelet therapy according to the cause of ARDS, are advocated in this area.

## Additional files

Additional file 1: PRISMA 2009 checklist. (DOC 64 kb)

Additional file 2: Study protocol. (DOCX 44 kb)

Additional file 3: Supplemental Tables. Studies excluded with reasons. Data regarding baseline patient characteristics, interventions and outcomes. Quality of the included studies. (DOCX 128 kb)

Additional file 4: Supplemental Figures. Risk of bias of the included studies and funnel plots. (DOCX 12877 kb)

## Abbreviations

AECC: American-European Consensus Conference
ARDS: Acute respiratory distress syndrome
GRADE: Grading of Recommendations Assessment, Development, and Evaluation
ICU: Intensive care unit
NOS: Newcastle-Ottawa Scale
OR: Odds ratio
PRISMA: Preferred Reporting Items for Systematic Reviews and Meta-Analyses
RCT: Randomized controlled clinical trial
