# Supplementary material for: The preventive effect of antiplatelet therapy in acute respiratory distress syndrome: a meta-analysis
Source: Crit Care. 2018 Mar 8;22:60. doi: 10.1186/s13054-018-1988-y (PMC5844104; doi:10.1186/s13054-018-1988-y)
Supplement: Supplementary file 2 — Study protocol. (DOCX 44 kb) [file 13054_2018_1988_MOESM2_ESM.docx]

# PROTOCOL

December 2016

## **Objectives**

To investigate whether antiplatelet therapy can reduce the incidence of newly-developed ARDS and mortality in patients at high risk of acute respiratory distress syndrome (ARDS)

## PICOS

Population: Adult patients at high risk of ARDS

Intervention: Antiplatelet therapy at any time or dose

Comparison: No antiplatelet therapy

Outcome: Newly-developed ARDS, mortality

Study design: Randomized control trial, observational cohort study, case-control study

## Search Strategy

Search databases: Cochrane Central Register of Controlled Trials (CENTRAL), Pubmed, Embase，Medline, Web of Science

**Pubmed**

#1 ARDS[Title/Abstract]

#2 "Respiratory Distress Syndrome, Adult"[Mesh]

#3 #1 OR #2

#4 "Platelet Aggregation Inhibitors"[Mesh]

#5 antiplatelet drugs

#6 aspirin

#7 clopidogrel

#8 prasugrel

#9 ticlopidine

#10 cilostazol

#11 dipyridamole

#12 tirofiban

#13 eptifibatide

#14 abciximab

#15 anagrelide

#16 ticagrelor

#17 vorapaxar

#18 atopaxar

#19 Pentoxifylline

#20 antiplatelet

#21 anti-platelet

#22 #4 OR #5 OR #6 OR #7 OR #8 OR #9 OR #10 OR #11 OR #12 OR #13 OR #14 OR # 15 OR #16 OR #17 OR #18 OR #19 OR #20 OR #21

#23 #3 AND #22

**EMBASE (Ovid version)**

#1 exp antithrombocytic agent/

#2 exp thrombocyte antibody/

#3 (antiplatelet drugs or aspirin or clopidogrel or prasugrel or ticlopidine or cilostazol or dipyridamole or tirofiban or eptifibatide or abciximab or anagrelide or ticagrelor or vorapaxar or atopaxar or pentoxifylline or antiplatelet or anti-platelet).af.

#4 #1 or #2 or #3

#5 exp adult respiratory distress syndrome/

#6 acute respiratory distress syndrome.af.

#7 ARDS.af.

#8 #5 or #6 or #7

#9 #4 and #8

**MEDLINE (Ovid version)**

#1 exp Respiratory Distress Syndrome, Adult/

#2 adult respiratory distress syndrome.af.

#3 ARDS.af.

#4 #1 or #2 or #3

#5 exp Platelet Aggregation Inhibitors/

#6 (antiplatelet drugs or aspirin or clopidogrel or prasugrel or ticlopidine or cilostazol or dipyridamole or tirofiban or eptifibatide or abciximab or anagrelide or ticagrelor or vorapaxar or atopaxar or pentoxifylline or antiplatelet or anti-platelet).af.

#7 #4 and #6

**CENTRAL**

#1 ARDS:ti,ab,kw

#2 MeSH descriptor: [Respiratory Distress Syndrome, Adult] explode all trees

#3 MeSH descriptor: [Platelet Aggregation Inhibitors] explode all trees

#4 'antiplatelet drugs' or 'aspirin' or 'clopidogrel' or 'prasugrel' or 'ticlopidine' or 'cilostazol' or 'dipyridamole' or 'tirofiban' or 'eptifibatide' or 'abciximab' or 'anagrelide' or 'ticagrelor' or 'vorapaxar' or 'atopaxar' or 'pentoxifylline' or 'antiplatelet' or 'anti-platelet'

#5 (#1 or #2) and (#3 or #4)

**Web of Science**

#1 TS=('antiplatelet drugs' OR 'aspirin' OR 'clopidogrel' OR 'prasugrel' OR 'ticlopidine' OR 'cilostazol' OR 'dipyridamole' OR 'tirofiban' OR 'eptifibatide' OR 'abciximab' OR 'anagrelide' OR 'ticagrelor' OR 'vorapaxar' OR 'atopaxar' OR 'pentoxifylline' OR 'antiplatelet' OR 'anti-platelet)

#2 TS=('acute respiratory distress syndrome' OR 'ARDS')

#3 #1 AND #2

## Inclusion Criteria

Inclusion of adult patients at high risk of ARDS at the time of presentation in hospital or intensive care unit (ICU)

Evaluation of antiplatelet therapy

Newly-developed ARDS reported

Randomized studies, observational cohort studies or case-control studies

Full text in English-language version available

In accordance with the latest definition of ARDS, Berlin Definition, patients previous reported as acute lung injury (ALI) will also be regarded as a subgroup of ARDS in this investigation. High-risk factors of ARDS, identified from previous research to be closely associated with the development of ARDS, are defined as sepsis, non-cardiogenic shock, trauma, high-risk surgery, aspiration, pneumonia, pancreatitis and massive transfusion[[1-4](#_ENREF_1)]. Antiplatelet therapy is defined as administration of one or more antiplatelet agents including irreversible cyclooxygenase (COX) inhibitor (aspirin), adenosine diphosphate inhibitors (clopidogrel, prasugrel, ticlopidine, and ticagrelor), phosphodiesterase inhibitors (cilostazol, anagrelide, Pentoxifylline), adenosine reuptake inhibitors (dipyridamole), glycoprotein IIb/IIIa inhibitors (tirofiban, eptifibatide, and abciximab), and protease activated receptor-1 antagonist (atopaxar, vorapaxar)[[5](#_ENREF_5)]. The age of population in the included study will be estimated with mean age and standard difference if it was not specifically stated in the study inclusion criteria.

## Exclusion Criteria

Inclusion of patients without risk of ARDS

Inclusion of patients with developed ARDS at the beginning of the study

Lack of a contrast group

## **Primary Outcome**

Incidence of newly-developed ARDS in patients at high risk of ARDS

## **Secondary Outcome**

Hospital and ICU mortality

## Selection of Studies

Two authors will independently screen titles and abstracts of searched records to select potentially eligible studies for full-text review. Any study identified as potentially eligible by either reviewer will be kept for full-text review. Authors will manually review the references of selected review articles to find other relevant studies. Two authors will separately review the full text of potentially relevant studies to identify eligible studies. All disagreements will be resolved by discussion with a third author.

## Data Extraction

A data collection form will be designed by the study team in Microsoft Excel. All data will be extracted and recorded by two authors independently. Arguments will be settled by discussion. Authors of studies will be contacted for further information as needed.

The following variables will be recorded:

*Study characteristics*: First author, publication year, study design, number of study center, location of study centers, the scale of the trial, study population, definition of antiplatelet therapy, definition of antiplatelet therapy, definition of ARDS

*Intervention*: Exposure time of antiplatelet therapy, type of antiplatelet therapy, administration and dose of antiplatelet therapy

*Baseline characteristics*: Number of patients in antiplatelet group and non-antiplatelet group, number of male, illness severity score, number of patients in different risk factors of ARDS

*Outcome*: Incidence of newly-developed ARDS, reported mortality

For observational studies, both adjusted and unadjusted odds ratio (OR) for incidence of newly-developed ARDS and mortality will be collected.

## Quality Assessment

**Risk of bias**

*Randomized studies:* Study methodological quality will be assessed with the Cochrane Collaboration Risk of Bias Tool[[6](#_ENREF_6)]. Each study will be evaluated as having a low risk, high risk, or unclear risk of bias in the following areas: (1) sequence generation, (2) allocation concealment, (3) blinding of participants/personnel/outcome assessors, (4) incomplete outcome data, (5) selective outcome reporting, and (6) other sources of bias[[6](#_ENREF_6)].

*Observational studies:* Quality will be assessed using the Newcastle-Ottawa Scale (NOS)[[7](#_ENREF_7)]. Two different versions of nine stars NOS for cohort and case-control studies will be applied accordingly. Each study will be evaluated on a 9-point scale in the domains of patient selection, comparability, exposure and outcome. We judge observational studies with NOS score of nine or eight stars as low risk bias, seven or six stars as median risk of bias, and below six stats as high risk of bias because criteria for quality of a study have not been established[[8](#_ENREF_8)].

**Quality of evidence**

Grading of Recommendations Assessment, Development, and Evaluation (GRADE) will be used to make judgments about quality of evidence for each outcome[[9](#_ENREF_9)]. GRADE presents a systematic and transparent framework for clarifying questions, determining the outcomes of interest, summarizing the evidence that addresses a question, and moving from the evidence to a recommendation or decision[[9](#_ENREF_9)]. We will assess the quality of evidence as high, moderate, low, or very low using GRADE profiler 3.6 (GRADEpro; McMaster University 2014, Hamilton, Canada)

## Data Analysis

Randomized and observational studies will be analyzed separately.

*Randomized studies*: A odds ratio (OR) with 95% confidence interval (CI) will be calculated for each study and combined using the number of patients with and without the outcome in exposed and non-exposed groups.

*Observational studies*: An adjusted OR with 95% confidence interval will be extracted from each study and combined. If the adjusted OR is not provided in the text or table, an OR will be calculated using the number of patients with and without the outcome in exposed and non-exposed groups. For studies that report multiple adjusted ORs, the OR adjusted for the greatest number of variables will be used in this meta-analysis.

Primary and secondary outcomes will be pooled with recorded data. Heterogeneity will be assessed with Q statistic and Higgens I^2^ test. Q statistic (P≥0.1) and I^2^-statistic (I^2^＜30%) will be regarded as low heterogeneity. Chi-square test will be used to combine data with 95% CI. A fixed model will be taken for data combination with low heterogeneity. Elsewise, a random effect model will be used. A two-tailed P value less than 0.05 will be considered as statistically significant.

Publication bias for the primary outcome will be assessed separately in randomized studies and observational studies using funnel plots and Egger’s test.

## Subgroup and Sensitivity Analyses

The primary outcome will be evaluated in definition of antiplatelet therapy (use aspirin only, use drug in combination with aspirin or others), the time of antiplatelet exposure (before hospitalization or after hospitalization), dose of antiplatelet agents, risk factors of ARDS, definition of ARDS (Berlin definition, the ACCE definition or others), size of populations (≤1000 or > 1000) and both in randomized and observational studies and the design of the studies(prospective, retrospective or case-control) in observational studies. Sensitivity analysis will be performed to investigate the robustness of our result of the primary outcome[[10](#_ENREF_10)]. We will repeat our analysis omitting one study at a time to assess whether any of included studies has a large effect on the result[[10](#_ENREF_10), [11](#_ENREF_11)].

All statistical analyses will be performed in STATA, version 13.0 (Stata Corporation, College Station, Texas).

## Reporting of Results

Results will be reported according to the Preferred Reporting Items for Systematic Reviews and Meta-Analyses (PRISMA) (<http://www.prisma-statement.org>)[[12](#_ENREF_12)].

## Reference

1. Bellani G, Laffey JG, Pham T *et al.* **Epidemiology, Patterns of Care, and Mortality for Patients With Acute Respiratory Distress Syndrome in Intensive Care Units in 50 Countries**. *JAMA.* 2016; **315**(8):788-800.

2. Hudson LD, Milberg JA, Anardi D *et al.* **Clinical risks for development of the acute respiratory distress syndrome**. *Am J Respir Crit Care Med.* 1995; **151**(2 Pt 1):293-301.

3. Gajic O, Dabbagh O, Park PK *et al.* **Early identification of patients at risk of acute lung injury: evaluation of lung injury prediction score in a multicenter cohort study**. *Am J Respir Crit Care Med.* 2011; **183**(4):462-70.

4. Ferguson ND, Frutos-Vivar F, Esteban A *et al.* **Clinical risk conditions for acute lung injury in the intensive care unit and hospital ward: a prospective observational study**. *Crit Care.* 2007; **11**(5):R96.

5. Mohananey D, Sethi J, Villablanca PA *et al.* **Effect of antiplatelet therapy on mortality and acute lung injury in critically ill patients: A systematic review and meta-analysis**. *Annals of Cardiac Anaesthesia.* 2016; **19**(4):626-37.

6. Higgins JP, Altman DG, Gotzsche PC *et al.* **The Cochrane Collaboration's tool for assessing risk of bias in randomised trials**. *BMJ.* 2011; **343**:d5928.

7. Wells GA, Shea BJ, O'Connell D *et al.* **The Newcastle–Ottawa Scale (NOS) for Assessing the Quality of Non-Randomized Studies in Meta-Analysis**. *Applied Engineering in Agriculture.* 2014; **18**(6):727-34.

8. Wang L, Li H, Gu X *et al.* **Effect of antiplatelet therapy on acute respiratory distress syndrome and mortality in critically ill patients: A meta-analysis**. *PLoS ONE.* 2016; **11**(5):e0154754.

9. Guyatt G, Oxman AD, Akl EA *et al.* **GRADE guidelines: 1. Introduction-GRADE evidence profiles and summary of findings tables**. *J Clin Epidemiol.* 2011; **64**(4):383-94.

10. Higgins JP, Green S. **Cochrane Handbook for Systematic Reviews of Interventions**. *Naunyn-Schmiedebergs Archiv für experimentelle Pathologie und Pharmakologie.* 2010; **2011**(14):S38.

11. Elmariah S, Mauri L, Doros G *et al.* **Extended duration dual antiplatelet therapy and mortality: a systematic review and meta-analysis**. *Lancet.* 2015; **385**(9970):792-8.

12. Moher D, Liberati A, Tetzlaff J *et al.* **Preferred reporting items for systematic reviews and meta-analyses: the PRISMA statement**. *PLoS Med.* 2009; **6**(7):e1000097.
