# Supplementary material for: The preventive effect of antiplatelet therapy in acute respiratory distress syndrome: a meta-analysis
Source: Crit Care. 2018 Mar 8;22:60. doi: 10.1186/s13054-018-1988-y (PMC5844104; doi:10.1186/s13054-018-1988-y)
Supplement: Supplementary file 3 — Supplemental Tables. Studies excluded with reasons. Data regarding baseline patient characteristics, interventions and outcomes. Quality of the included studies. (DOCX 128 kb) [file 13054_2018_1988_MOESM3_ESM.docx]

**Table S1. Studies Excluded after Full-text Review**

| Author, year | Reasons for exclusion |
| --- | --- |
| Brevard,2008[[1](#_ENREF_1)] | Case report study |
| Griffith,2005[[2](#_ENREF_2)] | Review article |
| Sarma,1990[[3](#_ENREF_3)] | Letter to editor |
| Al Harbi,2016[[4](#_ENREF_4)] | Incidence of newly-developed ARDS not reported；the risk of ARDS not clearly described |
| Tabrizi,2012[[5](#_ENREF_5)] | Incidence of newly-developed ARDS not reported; inclusion of patients younger than 18 years old |
| Zeni,1996[[6](#_ENREF_6)] | Incidence of newly-developed ARDS not reported |
| Bein,1996[[7](#_ENREF_7)] | Lack of a control group with non-antiplatelet therapy; incidence of newly-developed ARDS not reported; |
| Meyer,1998[[8](#_ENREF_8)] | Incidence of newly-developed ARDS not reported; inclusion of patient with developed ARDS at the beginning of the study; lack of a control group with non-antiplatelet therapy; |
| Bacher,1997[[9](#_ENREF_9)] | Lack of a control group with non-antiplatelet therapy; incidence of newly-developed ARDS not reported; |
| Elmer,2013[[10](#_ENREF_10)] | Irrelevant: association between ARDS and antiplatelet therapy not investigated |
| Bein,2011[[11](#_ENREF_11)] | Irrelevant: association between ARDS and antiplatelet therapy not investigated |
| Eisen,2012[[12](#_ENREF_12)] | Incidence of newly-developed ARDS not reported |
| Harr,2013[[13](#_ENREF_13)] | Incidence of newly-developed ARDS not reported |
| Otto,2013[[14](#_ENREF_14)] | Incidence of newly-developed ARDS not reported; data of a control group with non-antiplatelet therapy not reported |
| Losche,2012[[15](#_ENREF_15)] | Review article |
| Winning,2009[[16](#_ENREF_16)] | Incidence of newly-developed ARDS not reported |
| Winning,2010[[17](#_ENREF_17)] | The risk of ARDS of included patients not clearly described; incidence of newly-developed ARDS not reported |
| Tsai,2015[[18](#_ENREF_18)] | Incidence of newly-developed ARDS not reported |
| Gross,2013[[19](#_ENREF_19)] | Inclusion of patients without high risk of ARDS |
| Locker,1997[[20](#_ENREF_20)] | Investigate prostaglandin E1 which has various effects on ARDS |

**Table S2. Baseline Patient Characteristics of the Randomized Studies**

| Author/year | Study group | Sample size | Male No. (%) | Age (years)^a^ | Illness severity scores ^a^ | Mechanical ventilation No. (%) | Sepsis  No. (%) | Shock  No. (%) | Pneumonia  No. (%) | Aspiration  No. (%) | Trauma  No. (%) | High risk surgery  No. (%) | Pancreatitis  No. (%) | Massive transfusion  No. (%) |
| --- | --- | --- | --- | --- | --- | --- | --- | --- | --- | --- | --- | --- | --- | --- |
| Kor.2016[[21](#_ENREF_21)] | Intervention  Control | 195  195 | 107 (54.9)  96 (49.2) | 57.0 (44.0-67.0)  57.0 (47.0-68.0) | LIP 6.0(5.0-7.5)  LIP 5.5(4.5-7.5) | 39(20.0)^b^  29(14.9)^b^ | 150 (76.9)  153 (78.5) | 41 (21.0)  40 (20.5) | 120 (61.5)  116 (59.5) | 28 (14.4)  22 (11.3) | 9 (4.6)  15 (7.7) | 0  1 (0.5) | 1(0.5)  0 | -  - |
| Vincent,  1985[[22](#_ENREF_22)] | Intervention  control | 16  17 | -  - | -  - | -  - | -  - | 5 (31.25)  7 (41.2) | 16 (100)  17 (100) | -  - | -  - | 5 (31.3)  4 (23.5) | -  - | -  - | -  - |

^a^ Continuous variables reported as mean (standard deviation) or median (interquartile range) unless otherwise indicated.

^b^ Mechanical ventilation reported in randomized day.

**Table S3. Intervention Characteristics of Randomized Studies**

| Author, year | Exposure time of antiplatelet therapy | Antiplatelet drugs | Administration and dose of antiplatelet drugs |
| --- | --- | --- | --- |
| Kor, 2016[[21](#_ENREF_21)] | Within 24 hours after presentation to the hospital | Aspirin | A 325mg loading dose in day one followed by 81mg once daily to day 7 |
| Vincent,1985[[22](#_ENREF_22)] | After an episode of non-cardiogenic circulatory shock | Dipyridamole | Constant iv infusion of 3mg/kg•24h until discharged from ICU or after 7 days |

Abbreviation: ICU intensive care unit, iv intravenous injection

**Table S4. Outcome Data of the Randomized Studies**

| Author, year | Study group | Sample size | ARDS  No. (%)^a^ | ICU mortality  No. (%) | 28-day mortality No. (%) | Hospital mortality No. (%) | Other reported mortality No. (%) |
| --- | --- | --- | --- | --- | --- | --- | --- |
| Kor,2016[[21](#_ENREF_21)] | Intervention  Control | 195  195 | 20 (10.3)  17 (8.7) | -  - | 18 (9.2)  18 (9.2) | 14 (7.2)  14 (7.2) | 1-year 45 (23.1)  1-year 44 (22.6) |
| Vincent,1985[[22](#_ENREF_22)] | Intervention  Control | 16  17 | 2 (12.5)  0 (0.0) | -  - | -  - | 6 (37.5)  4 (23.5) | -  - |

1. ARDS within 7 days.

**Table S5. GREAD Evidence Profile of the Randomized studies**
**Question:** Antiplatelet versus non-antiplatelet for patients at high risk of ARDS
**Settings:** Hospital and/or ICU

| **Quality assessment** | | | | | | | **No of patients** | | **Effect** | | **Quality** | **Importance** |
| --- | --- | --- | --- | --- | --- | --- | --- | --- | --- | --- | --- | --- |
|  |  |  |  |  |  |  |  |  |  |  |  |  |
| **No of studies** | **Design** | **Risk of bias** | **Inconsistency** | **Indirectness** | **Imprecision** | **Other considerations** | **Antiplatelet versus non-antiplatelet** | **Control** | **Relative (95% CI)** | **Absolute** |  |  |
| **Newly-developed ARDS** | | | | | | | | | | | | |
| 2 | randomized trials | serious^1^ | no serious inconsistency | no serious indirectness | serious^2^ | none | 22/211  (10.4%) | 17/212  (8%) | OR 1.29 (0.66 to 2.5) | 21 more per 1000 (from 26 fewer to 99 more) | ⊕⊕OO LOW | CRITICAL |
|  |  |  |  |  |  |  |  | 4.4% |  | 12 more per 1000 (from 15 fewer to 59 more) |  |  |
| **Hospital mortality** | | | | | | | | | | | | |
| 2 | randomized trials | serious^1^ | no serious inconsistency | no serious indirectness | serious^2^ | none | 20/211  (9.5%) | 18/212  (8.5%) | OR 1.15 (0.58 to 2.27) | 12 more per 1000 (from 34 fewer to 89 more) | ⊕⊕OO LOW | CRITICAL |
|  |  |  |  |  |  |  |  | 15.4% |  | 19 more per 1000 (from 59 fewer to 138 more) |  |  |

^1^ Two of three randomized studies did not state the randomization methods and allocation concealments. (Downgraded for serious risk of bias.)
^2^ The confidence interval for combined OR included no effect and important harm. (Downgraded for serious imprecision.)

**Table S6. Baseline Patient Characteristics of the Observational Studies**

| Author, year | Study group | Sample size | Male  No. (%) | Age (years)^a^ | Illness severity scores ^a^ | Mechanical ventilation No. (%) | Sepsis  No. (%) | Shock  No. (%) | Pneumonia  No. (%) | Aspiration  No. (%) | Trauma  No. (%) | High risk surgery  No. (%) | Pancreatitis  No. (%) | Massive transfusion  No. (%) |
| --- | --- | --- | --- | --- | --- | --- | --- | --- | --- | --- | --- | --- | --- | --- |
| Mazzeffi,2015 [[23](#_ENREF_23)] | Anti  Non-anti | 181  194 | 99 (55.0)  114 (58.8) | 72 (64-80)  57.0 (47.0-68.0) | -  - | -  - | -  - | -  - | -  - | -  - | -  - | 181 (100)  194 (100) | -  - | -  - |
| Chen,2015[[24](#_ENREF_24)] | Anti  Non-anti | 287  862 | 167 (58.2)  464 (53.8) | 67 (61-74)  58 (50-65) | -  - | -  - | -  - | -  - | -  - | -  - | -  - | -  - | -  - | -  - |
| Valerio,2013 [[25](#_ENREF_25)] | Anti  Non-anti | 272  379 | 160 (58.8)  202 (53.3) | 75.2 (65.2-82.5)  65.8 (53.2-78.3) | APACHEⅢ 57.5 (46.0-74.8)  APACHEⅢ 55.0 (42.0-68.0) | -  - | 272 (100)  379 (100) | -  - | -  - | -  - | -  - | -  - | -  - | -  - |
| Kor,2011[[26](#_ENREF_26)] | Anti  Non-anti | 976  2879 | 541 (55.4)  1574 (54.7) | 70 (59-81)  51 (38-66) | APACHEⅡ 12 (8-16)  LIPS 2.5 (1.5-4.0)  APACHEⅡ 9 (5-14)  LIPS 2.5 (1.5-4.0) | -  - | 443 (45.4)  1306 (45.4) | 91 (9.3)  274 (9.5) | 391 (40.1)  834 (29.0) | 52 (5.3)  151 (5.2) | 170 (17.4)  768 (26.7) | -  - | 68 (7.0)  240 (8.3) | -  - |
| Erlich,2011[[27](#_ENREF_27)] | Anti  Non-anti | 79  82 | 42 (53.2)  44 (53.7) | 77(12)  66(19) | APACHEⅢ 46 (34-57)  LIPS 3 (1.5-4.0)  APACHEⅢ 39 (27-54)  LIPS 3 (1.5-4.0) | 8 (10.1)  11 (13.4) | 19 (24.1)  25 (30.5) | 40 (50.6)  47 (57.3) | 30 (38.0)  25 (30.5) | 11 (13.9)  8 (9.8) | 4 (5.1)  4 (4.9) | -  - | 2 (2.5)  2 (2.4) | -  - |
| Ahmed,2014 [[28](#_ENREF_28)] | Case  Control | 414  414 | 245 (59)  245 (59) | 66 (17)  66 (17) | LIPS 2 (1-3)  LIPS 2 (1-3) | -  - | 121 (29)  121 (29) | 75 (18)  49 (12) | 83 (20)  103 (25) | 14 (3)  14 (3) | 24 (6)  16 (4) | 77 (19)  77 (19) | 10 (2)  14 (3) | -  - |
| Tuinman,2012 [[29](#_ENREF_29)] | Case  Control | 109  109 | -  - | -  - | APACHEⅡ 22 (8)  APACHEⅡ 19 (8) | 94 (86)  80 (73) | 36 (33)  20 (18) | -  - | -  - | -  - | -  - | 32 (31)  36 (33) | -  - | 37 (34)  16 (15) |

^a^ Continuous variables reported as mean (standard deviation) or median (interquartile range) unless otherwise indicated.

Abbreviations: LIPS Lung Injury Prediction Score, APACHE Acute Physiology and Chronic Health Evaluation, Anti antiplatelet therapy, Non-anti non-antiplatelet therapy

**Table S7. Intervention Characteristics of the Observational Studies**

| Author, year | Exposure time of antiplatelet therapy | Antiplatelet drugs | Administration and dose of antiplatelet drugs |
| --- | --- | --- | --- |
| Mazzeffi,2015[[23](#_ENREF_23)] | Preoperative use (within 5 days of surgery) | Aspirin | Preoperative aspirin was taken by 181 patients (48.3%) with the majority of patients taking 81 mg/d (72%). |
| Chen,2015[[24](#_ENREF_24)] | Prehospital aspirin use | Aspirin | 92 (31%) patients were taking 325mg per day. 184 (64%) patients were taking 81mg per day. In 11 patients the dose was not available. |
| Valerio,2013[[25](#_ENREF_25)] | Documentation of use or administration of antiplatelet therapy at the time of ICU admission | Aspirin, clopidogrel | The main antiplatelet drug used was aspirin as a single drug (88.6%) or in combination with clopidogrel (9.9%). Only 1.5% patients used clopidogrel. Antiplatelet therapy was discontinued in 48 (17.6%) of the patients who received it before ICU admission. Aspirin dose, mg, median (IQR) 81 (81-81). |
| Kor,2011[[26](#_ENREF_26)] | Before hospitalization | Aspirin | Not reported. |
| Erlich,2011[[27](#_ENREF_27)] | Documentation  of use or administration in the medical record of antiplatelet therapy  at the time of hospital admission | Aspirin, clopidogrel bisulfate, anagrelide | 68 patients were taking an ASA-containing medication alone, 3 patients were receiving clopidogrel bisulfate alone, and 1 patient was receiving the antiplatelet agent anagrelide. 7 patients were being treated with both ASA and clopidogrel bisulfate. |
| Ahmed,2014[[28](#_ENREF_28)] | During the at-risk period | Aspirin, abciximab, cilostazol, clopidogrel, dipyridamole, eptifibatide, ticlopidine | Not reported. |
| Tuinman,2012[[29](#_ENREF_29)] | At the time of ICU admission | Aspirin, clopidogrel | All patients used aspirin with an average dose of 80 or 100 mg once a day, 2 patients were also taking clopidogrel. |

Abbreviation: ICU intensive care unit, ASA acetylsalicylic acid

**Table S8. Outcome Data of the Observational Studies**

| Author, year | Study group | Sample size | ARDS | ICU mortality  No. (%) | 28-day mortality No. (%) | Hospital mortality No. (%) | Other reported mortality No. (%) |
| --- | --- | --- | --- | --- | --- | --- | --- |
| Mazzeffi,2015[[23](#_ENREF_23)] | Anti  Non-anti | 181  194 | 9 (5.0)  12 (6.7) | -  - | -  - | -  - | -  - |
| Chen,2015[[24](#_ENREF_24)] | Anti  Non-anti | 287  862 | 77 (27)  284(33) | -  - | -  - | Adjust OR 0.697 |  |
| Valerio,2013[[25](#_ENREF_25)] | Anti  Non-anti | 272  379 | 57 (21.0)  132 (34.8) | 26 (9.6)  46 (12.1) | -  - | 57 (21.0)  98 (25.9) | -  - |
| Kor,2011[[26](#_ENREF_26)] | Anti  Non-anti | 976  2879 | 44 (4.5)  196 (6.8) | 39 (4.0)  134 (4.7) | -  - | 63 (6.5)  180 (6.3) | -  - |
| Erlich,2011[[27](#_ENREF_27)] | Anti  Non-anti | 79  82 | 10 (12.7)  23 (28.1) | 9 (11.4)  9 (11.0) | -  - | 10 (12.7)  13 (15.9) | -  - |
| Ahmed,2014[[28](#_ENREF_28)] | Case  Control | 414  414 | -  - | -  - | -  - | -  - | -  - |
| Tuinman,2012[[29](#_ENREF_29)] | Case  Control | 109  109 | -  - | -  - | -  - | -  - | -  - |

Abbreviations: Anti antiplatelet therapy, Non-anti non-antiplatelet therapy

**Table S9. Risk of Bias in the Observational Studies**

| **Cohort studies** | | | | | | | | | |
| --- | --- | --- | --- | --- | --- | --- | --- | --- | --- |
| Study | Selection | | | | Comparability | Outcome | | | Total score |
|  | Exposed Cohort | Non-exposed Cohort | Ascertainment of exposure | Outcome of interest |  | Assessment of outcome | Length of follow-up | Adequacy of follow-up |  |
| Mazzeffi,2015[[23](#_ENREF_23)] | * | * | * | * | ** | * | 0 | 0 | 7 |
| Chen,2015[[24](#_ENREF_24)] | * | * | * | * | ** | * | 0 | 0 | 7 |
| Valerio,2013[[25](#_ENREF_25)] | * | * | * | 0 | ** | * | 0 | 0 | 6 |
| Kor,2011[[26](#_ENREF_26)] | * | * | * | * | ** | * | 0 | 0 | 7 |
| Erlich,2011[[27](#_ENREF_27)] | * | * | * | * | ** | * | * | 0 | 8 |
| **Case-control studies** | | | | | | | | | |
| Study | Selection | | | | Comparability | Exposure | | | Total score |
|  | Definition adequate | Representativeness | Selection of Controls | Definition of Controls |  | Ascertainment of exposure | Ascertainment for cases and controls | Non-Response rate |  |
| Ahmed,2014[[28](#_ENREF_28)] | * | * | * | * | 0 | * | * | 0 | 6 |
| Tuinman,2012 [[29](#_ENREF_29)] | * | * | * | * | ** | * | * | 0 | 8 |

**Table S10. GREAD Evidence Profile of the Cohort Studies**
**Question:** Should Antiplatelet therapy be used for patients at high risk of ARDS?
**Settings:** Hospital and/ICU

| **Quality assessment** | | | | | | | **No of patients** | | **Effect** | | **Quality** | **Importance** |
| --- | --- | --- | --- | --- | --- | --- | --- | --- | --- | --- | --- | --- |
|  |  |  |  |  |  |  |  |  |  |  |  |  |
| **No of studies** | **Design** | **Risk of bias** | **Inconsistency** | **Indirectness** | **Imprecision** | **Other considerations** | **Antiplatelet therapy** | **Control** | **Relative (95% CI)** | **Absolute** |  |  |
| **Newly-developed ARDS** | | | | | | | | | | | | |
| 5 | observational studies | no serious risk of bias | no serious inconsistency | no serious indirectness | no serious imprecision | none | 197/1795  (11%) | 647/4396  (14.7%) | OR 0.63 (0.52 to 0.75) | 49 fewer per 1000 (from 33 fewer to 65 fewer) | ⊕⊕OO LOW | CRITICAL |
|  |  |  |  |  |  |  |  | 28.1% |  | 83 fewer per 1000 (from 54 fewer to 112 fewer) |  |  |
| **Hospital mortality^1^** | | | | | | | | | | | | |
| 4 | observational studies | no serious risk of bias | no serious inconsistency | no serious indirectness | no serious imprecision | none | 179/1434  (12.5%) | 490/4202  (11.7%) | not pooled^1^ | not pooled^1^ | ⊕⊕OO LOW | CRITICAL |
|  |  |  |  |  |  |  |  | 19.5% |  | not pooled |  |  |
| **ICU mortality** | | | | | | | | | | | | |
| 3 | observational studies | no serious risk of bias | no serious inconsistency | no serious indirectness | serious^2^ | none | 74/1327  (5.6%) | 189/3340  (5.7%) | OR 0.84 (0.63 to 1.11) | 9 fewer per 1000 (from 20 fewer to 6 more) | ⊕OOO VERY LOW | CRITICAL |
|  |  |  |  |  |  |  |  | 11% |  | 16 fewer per 1000 (from 38 fewer to 11 more) |  |  |

^1^ The data of both groups were not accessible in Chen and his colleagues' study and therefore it failed to calculate assumed risks and corresponding risks.
^2^ The confidence interval for combined OR included no effect and important harm.(Downgraded for serious imprecision.)

**Table S11. GREAD Evidence Profile of the Case-control Studies**
**Question:** Should Antiplatelet therapy be used for patients at high risk of ARDS?
**Settings:** Hospital and/or ICU

| **Quality assessment** | | | | | | | **No of patients** | | **Effect** | | **Quality** | **Importance** |
| --- | --- | --- | --- | --- | --- | --- | --- | --- | --- | --- | --- | --- |
|  |  |  |  |  |  |  |  |  |  |  |  |  |
| **No of studies** | **Design** | **Risk of bias** | **Inconsistency** | **Indirectness** | **Imprecision** | **Other considerations** | **Antiplatelet therapy** | **Control** | **Relative (95% CI)** | **Absolute** |  |  |
| **Newly-develop ARDS** | | | | | | | | | | | | |
| 2 | observational studies^1^ | no serious risk of bias | no serious inconsistency | no serious indirectness | no serious imprecision | none | 523 cases 523 controls | | OR 0.90 (0.85 to 0.95) | - | ⊕⊕OO LOW |  |
|  |  |  |  |  |  |  |  | 0.0% |  | - |  |  |

^1^ Case-control

## References

1. Brevard S, Weintraub SL, Bronaugh H *et al.* **Effects of epoprostenol on pulmonary hypertension after pneumonectomy for trauma**. *Journal of Trauma - Injury, Infection and Critical Care.* 2008; **64**(2):496-9.

2. Griffiths MJD, Evans TW. **Inhaled nitric oxide therapy in adults**. *N Engl J Med.* 2005; **353**(25):2683-95.

3. Sarma PS. **Pentoxifylline in septic shock**. *Postgrad Med J.* 1990; **66**(781):980-1.

4. Al Harbi SA, Tamim HM, Al-Dorzi HM *et al.* **Association between aspirin therapy and the outcome in critically ill patients: a nested cohort study**. *BMC Pharmacol Toxicol.* 2016;17:5.

5. Tabrizi MB, Schinco MA, Tepas JJ *et al.* **Inhaled epoprostenol improves oxygenation in severe hypoxemia**. *Journal of Trauma and Acute Care Surgery.* 2012; **73**(2):503-6.

6. Zeni F, Pain P, Vindimian M *et al.* **Effects of pentoxifylline on circulating cytokine concentrations and hemodynamics in patients with septic shock: results from a double-blind, randomized, placebo-controlled study**. *Crit Care Med.* 1996; **24**(2):207-14.

7. Bein T, Metz C, Keyl C *et al.* **Cardiovascular and pulmonary effects of aerosolized prostacyclin administration in severe respiratory failure using a ventilator nebulization system**. *Journal of Cardiovascular Pharmacology.* 1996; **27**(4):583-6.

8. Meyer J, Theilmeier G, Van Aken H *et al.* **Inhaled prostaglandin E1 for treatment of acute lung injury in severe multiple organ failure**. *Anesth Analg.* 1998; **86**(4):753-8.

9. Bacher A, Mayer N, Klimscha W *et al.* **Effects of pentoxifylline on hemodynamics and oxygenation in septic and nonseptic patients**. *Crit Care Med.* 1997; **25**(5):795-800.

10. Elmer J, Hou P, Wilcox SR *et al.* **Acute Respiratory Distress Syndrome After Spontaneous Intracerebral Hemorrhage**. *Crit Care Med.* 2013; **41**(8):1992-2001.

11. Bein T, Zimmermann M, Philipp A *et al.* **Addition of Acetylsalicylic Acid to Heparin for Anticoagulation Management During Pumpless Extracorporeal Lung Assist**. *Asaio Journal.* 2011; **57**(3):164-8.

12. Eisen DP, Reid D, McBryde ES. **Acetyl salicylic acid usage and mortality in critically ill patients with the systemic inflammatory response syndrome and sepsis**. *Crit Care Med.* 2012; **40**(6):1761-7.

13. Harr JN, Moore EE, Johnson J *et al.* **Antiplatelet therapy is associated with decreased transfusion-associated risk of lung dysfunction, multiple organ failure, and mortality in trauma patients**. *Crit Care Med.* 2013; **41**(2):399-404.

14. Otto GP, Sossdorf M, Boettel J *et al.* **Effects of low-dose acetylsalicylic acid and atherosclerotic vascular diseases on the outcome in patients with severe sepsis or septic shock**. *Platelets.* 2013; **24**(6):480-5.

15. Losche W, Boettel J, Kabisch B *et al.* **Do aspirin and other antiplatelet drugs reduce the mortality in critically ill patients?** *Thrombosis.* 2012; **2012**:720254.

16. Winning J, Reichel J, Eisenhut Y *et al.* **Anti-platelet drugs and outcome in severe infection: clinical impact and underlying mechanisms**. *Platelets.* 2009; **20**(1):50-7.

17. Winning J, Neumann J, Kohl M *et al.* **Antiplatelet drugs and outcome in mixed admissions to an intensive care unit**. *Crit Care Med.* 2010; **38**(1):32-7.

18. Tsai MJ, Ou SM, Shih CJ *et al.* **Association of prior antiplatelet agents with mortality in sepsis patients: a nationwide population-based cohort study**. *Intensive Care Med.* 2015; **41**(5):806-13.

19. Gross AK, Dunn SP, Feola DJ *et al.* **Clopidogrel treatment and the incidence and severity of community acquired pneumonia in a cohort study and meta-analysis of antiplatelet therapy in pneumonia and critical illness**. *J Thromb Thrombolysis.* 2013; **35**(2):147-54.

20. Locker GJ, Staudinger T, Knapp S *et al.* **Prostaglandin E1 inhibits platelet decrease after massive blood transfusions during major surgery: influence on coagulation cascade?** *J Trauma.* 1997; **42**(3):525-31.

21. Kor DJ, Carter RE, Park PK *et al.* **Effect of Aspirin on Development of ARDS in At-Risk Patients Presenting to the Emergency Department The LIPS-A Randomized Clinical Trial**. *JAMA.* 2016; **315**(22):2406-14.

22. Vincent JL, Brimioulle S, Berre J *et al.* **Prevention of the adult respiratory distress syndrome with dipyridamole**. *Crit Care Med.* 1985; **13**(10):783-5.

23. Mazzeffi M, Kassa W, Gammie J *et al.* **Preoperative aspirin use and lung injury after aortic valve replacement surgery: A retrospective cohort study**. *Anesth Analg.* 2015; **121**(2):271-7.

24. Chen W, Janz DR, Bastarache JA *et al.* **Prehospital aspirin use is associated with reduced risk of acute respiratory distress syndrome in critically ill patients: A propensity-adjusted analysis**. *Crit Care Med.* 2015; **43**(4):801-7.

25. Valerio-Rojas JC, Jaffer IJ, Kor DJ *et al.* **Outcomes of severe sepsis and septic shock patients on chronic antiplatelet treatment: a historical cohort study**. *Crit Care Res Pract. 2013;2013:782573.*

26. Kor DJ, Erlich J, Gong MN *et al.* **Association of prehospitalization aspirin therapy and acute lung injury: Results of a multicenter international observational study of at-risk patients**. *Crit Care Med.* 2011; **39**(11):2393-400.

27. Erlich JM, Talmor DS, Cartin-Ceba R *et al.* **Prehospitalization antiplatelet therapy is associated with a reduced incidence of acute lung injury: A population-based cohort study**. *Chest.* 2011; **139**(2):289-95.

28. Ahmed AH, Litell JM, Malinchoc M *et al.* **The role of potentially preventable hospital exposures in the development of acute respiratory distress syndrome: A population-based study**. *Crit Care Med.* 2014; **42**(1):31-9.

29. Tuinman PR, Vlaar AP, Binnenkade JM *et al.* **The effect of aspirin in transfusion-related acute lung injury in critically ill patients**. *Anaesthesia.* 2012; **67**(6):594-9.
