# Supplementary material for: The preventive effect of antiplatelet therapy in acute respiratory distress syndrome: a meta-analysis
Source: Crit Care. 2018 Mar 8;22:60. doi: 10.1186/s13054-018-1988-y (PMC5844104; doi:10.1186/s13054-018-1988-y)
Supplement: Supplementary file 4 — Supplemental Figures. Risk of bias of the included studies and funnel plots. (DOCX 12877 kb) [file 13054_2018_1988_MOESM4_ESM.docx]

**Figure S1. Risk of Bias Summary of the Randomized Studies**


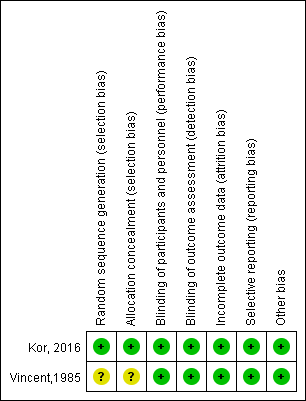


**Figure S2. Risk of Bias of Randomized Studies – Over All**


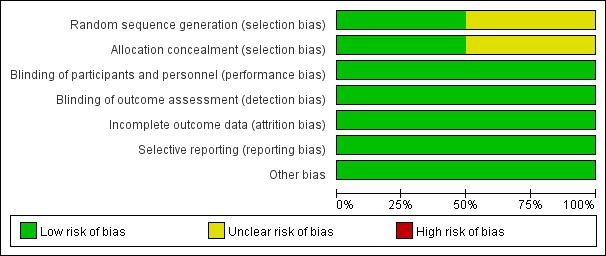


**Figure S3. The Effect of Antiplatelet Therapy on Hospital Mortality Based on the Randomized Studies**


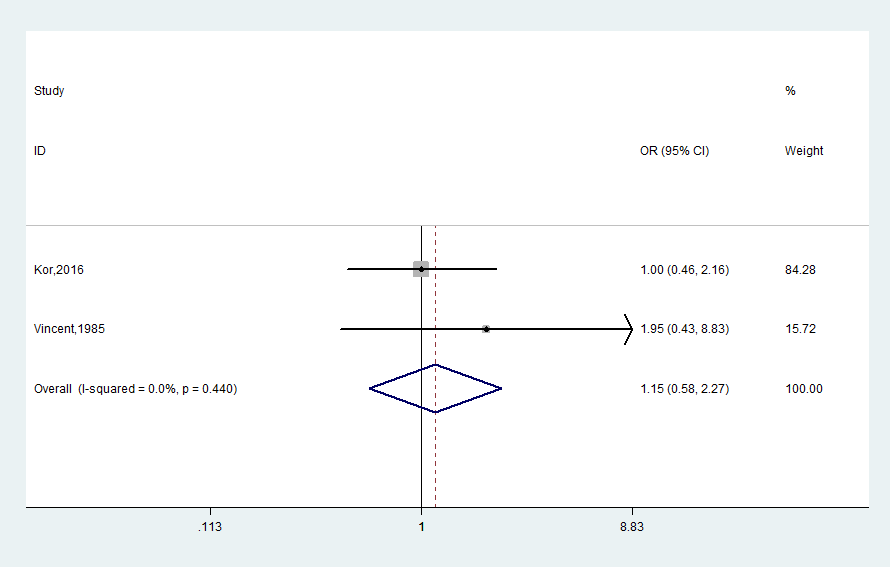


**Figure S4. The Effect of Antiplatelet Therapy on Hospital Mortality Based on the Observational Studies**


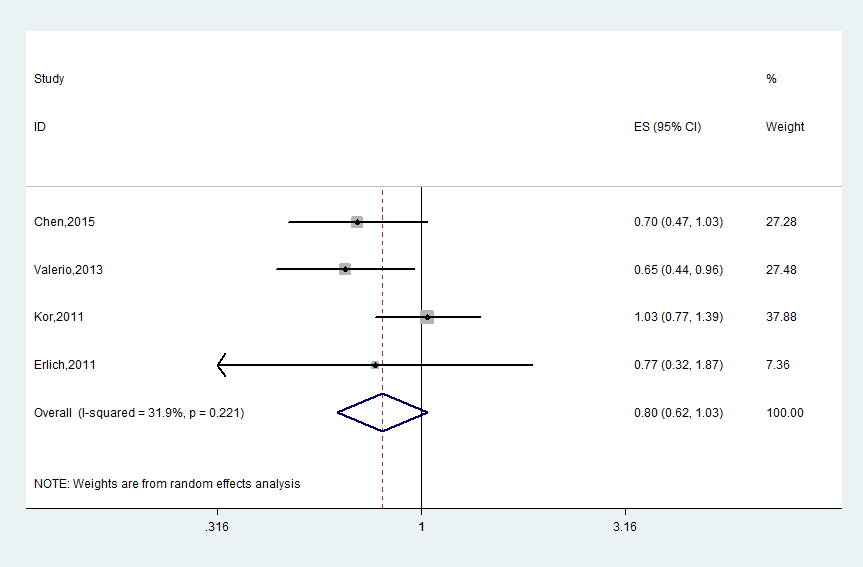


**Figure S5. The Effect of Antiplatelet Therapy on ICU Mortality Based on the Observational Studies**


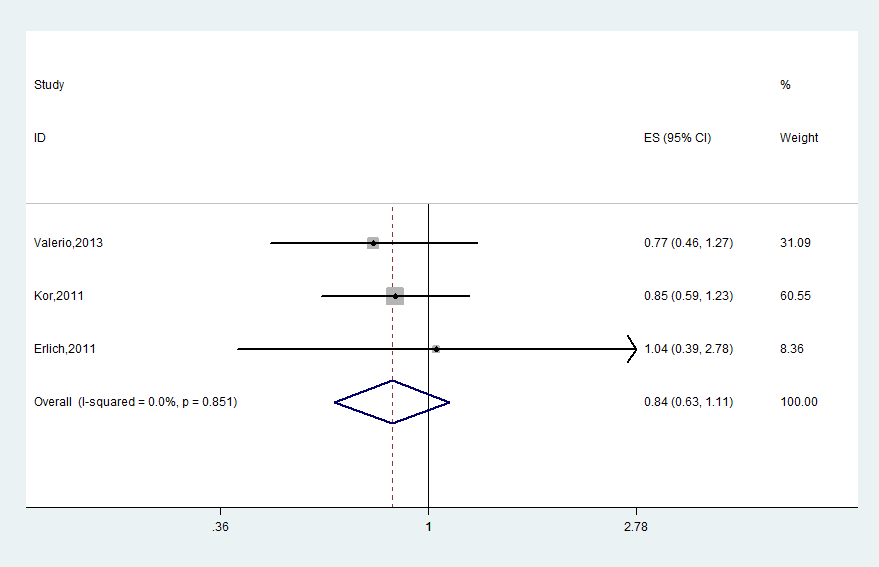


**Figure S6. The Subgroup Analysis of the Observational Studies by the Definition of Antiplatelet Therapy**


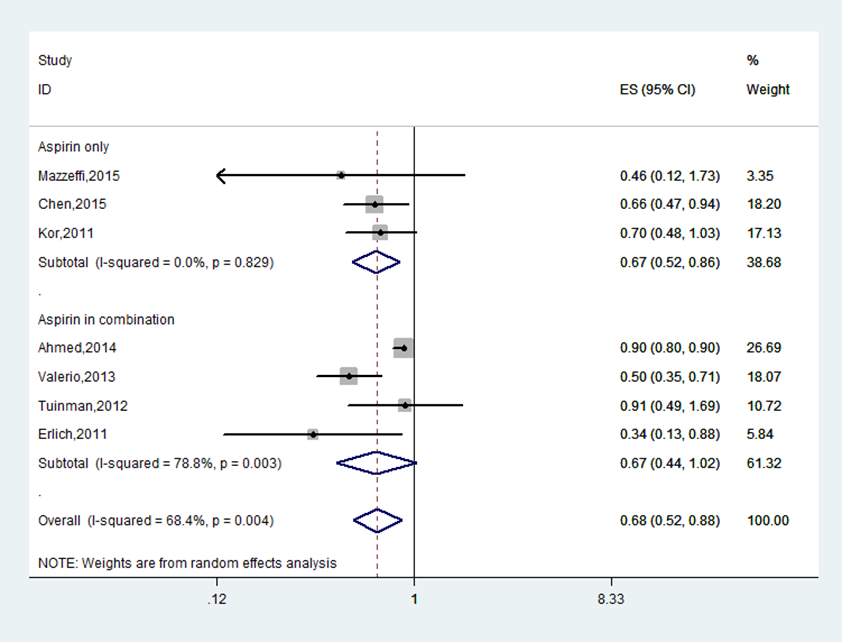


**Figure S6. The Subgroup Analysis of the Observational Studies by the Timing of Antiplatelet Therapy**


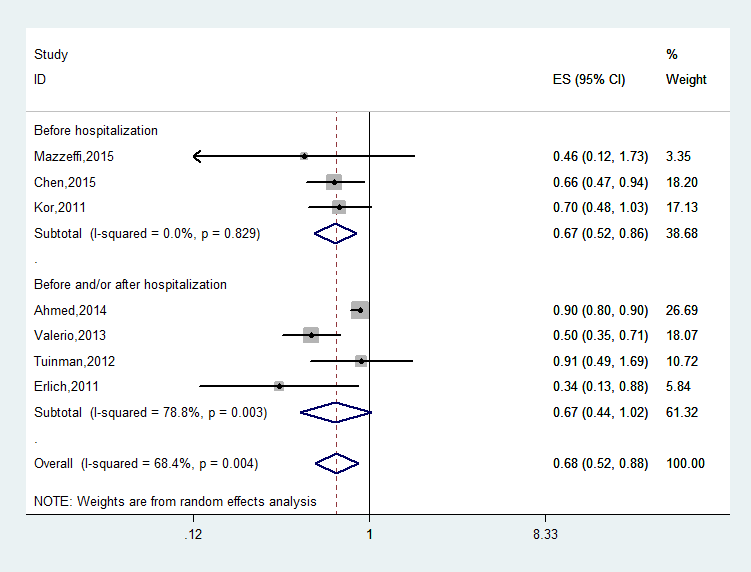


**Figure S7. The Subgroup Analysis of the Observational Studies by the Definition of ARDS**


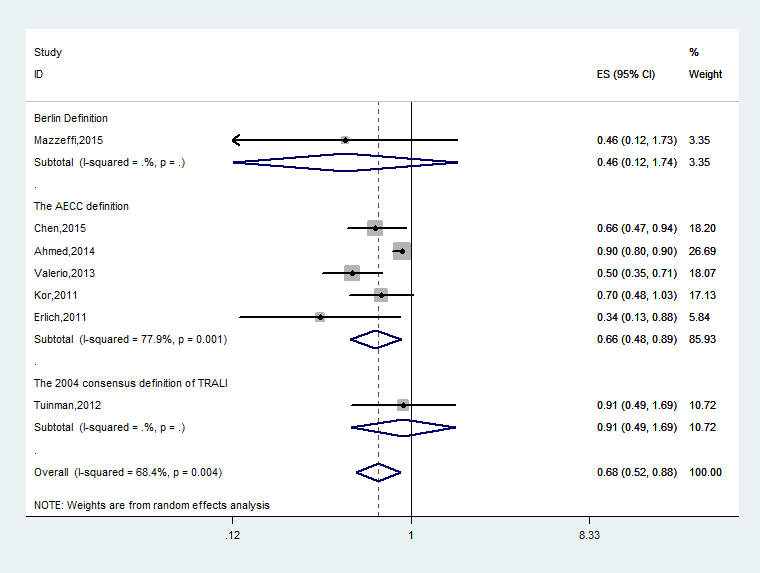


**Figure S8. The Subgroup Analysis of the Observational Studies by the Size of Population**


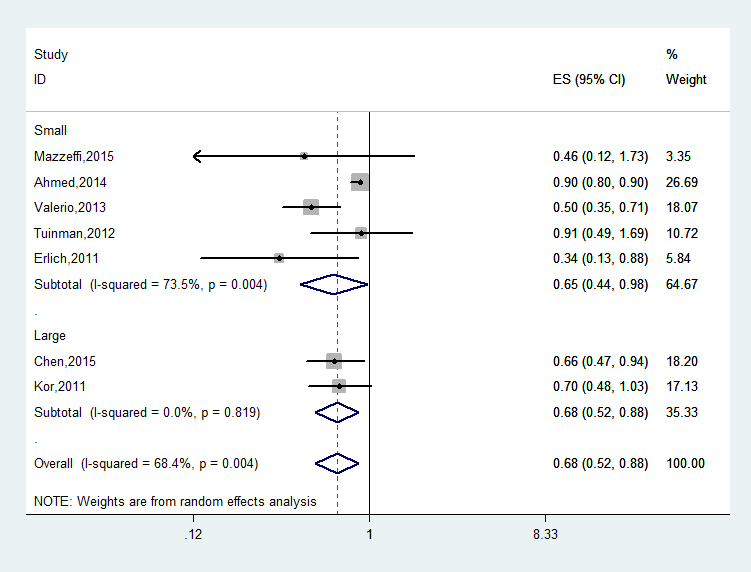


**Figure S9. The Subgroup Analysis of the Observational Studies by Study Design**


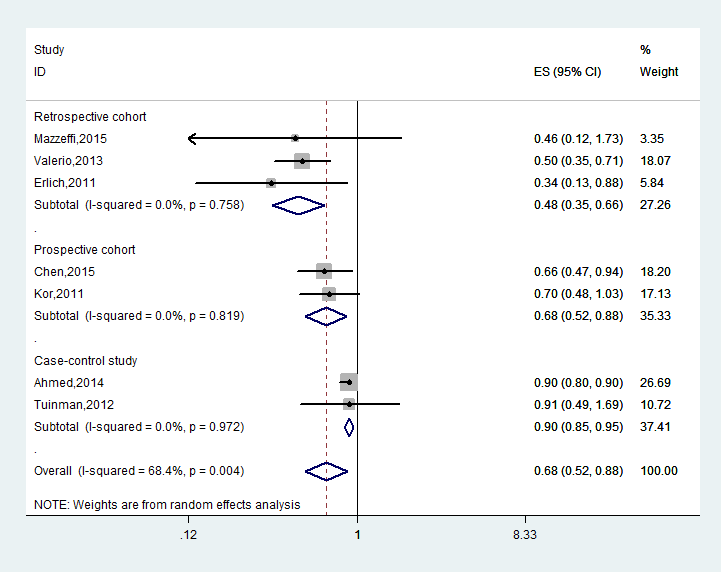


**Figure S10. The Subgroup Analysis of the Observational Studies by Inclusion of Sepsis Patients or not**


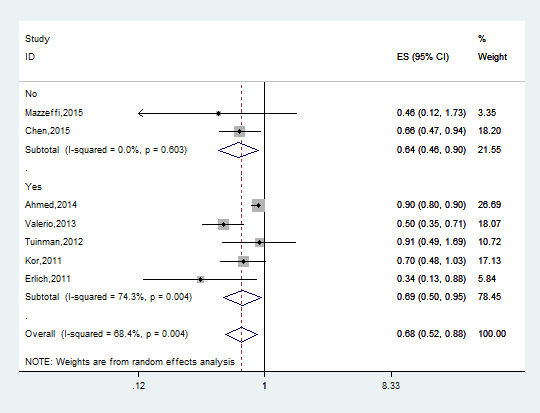


**Figure S11. The Subgroup Analysis of the Observational Studies by Inclusion of Shock Patients or not**


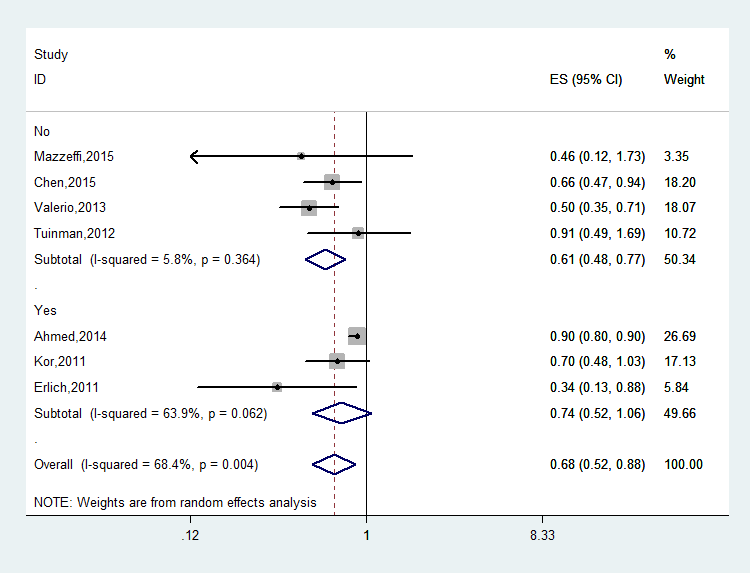


**Figure S12. The Subgroup Analysis of the Observational Studies by Inclusion of Pneumonia Patients or not**


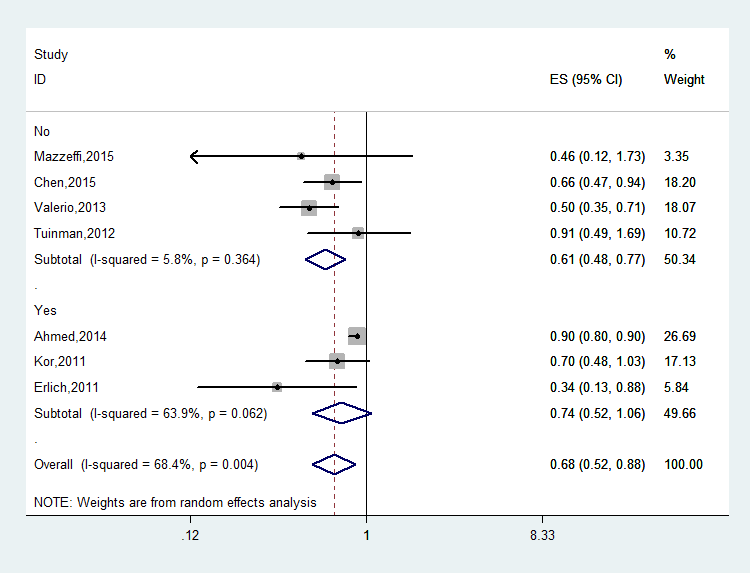


**Figure S13. Subgroup Analysis of the Observational Studies by Inclusion of Aspiration Patients or not**


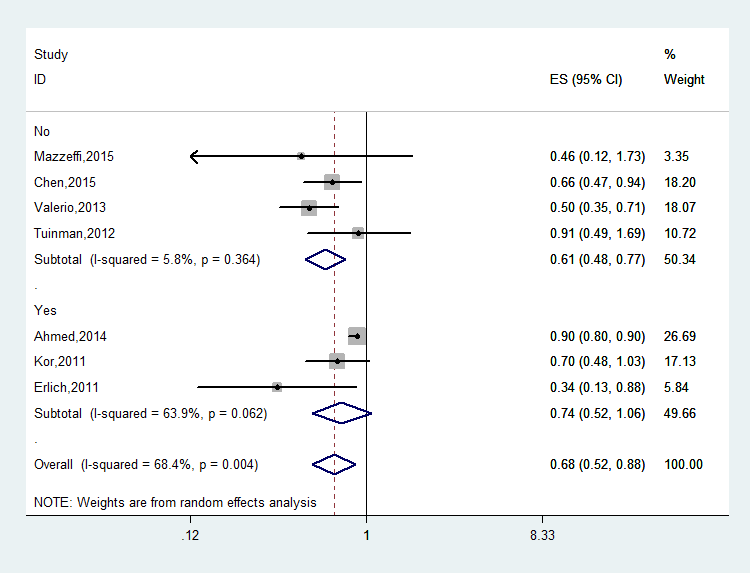


**Figure S14. The Subgroup Analysis of the Observational Studies by Inclusion of Trauma Patients or not**


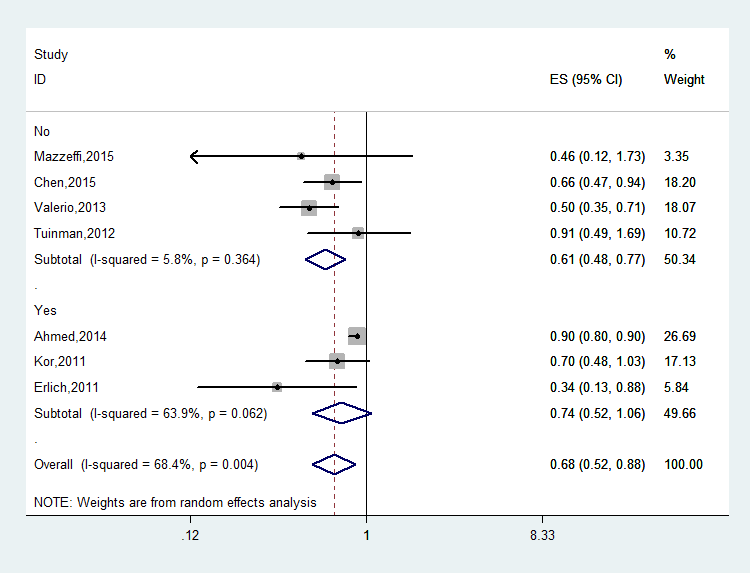


**Figure S15. The Subgroup Analysis of the Observational Studies by Inclusion of High-risk Surgical Patients or not**


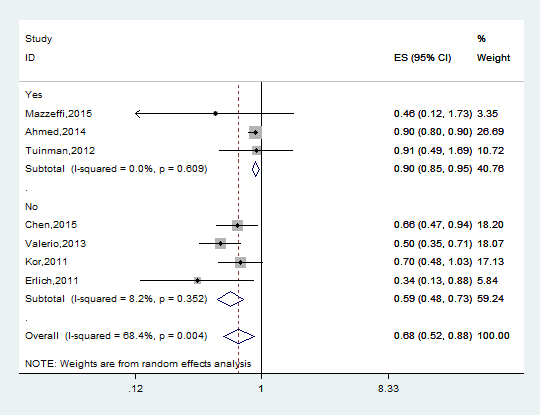


**Figure S16. The Subgroup Analysis of the Observational Studies by Inclusion of Pancreatitis Patients or not**


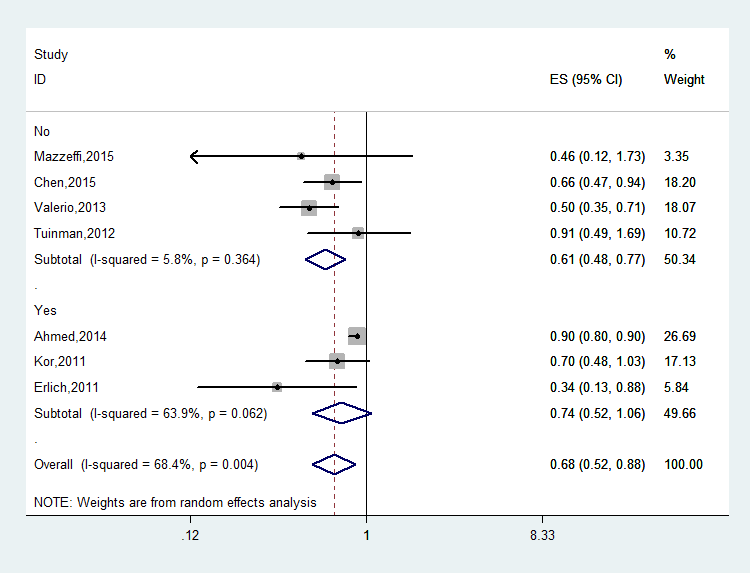


**Figure S17. The Subgroup Analysis of the Observational Studies by Inclusion of Massive Transfusion Patients or not**


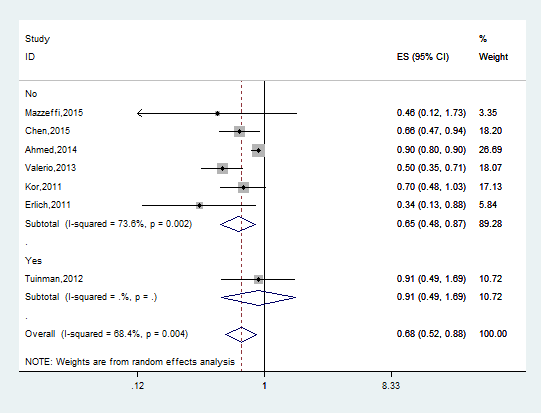


**Figure S18. The Effect of Antiplatelet therapy in Newly-developed ARDS after Study Conducted by Ahmed et al Removed**


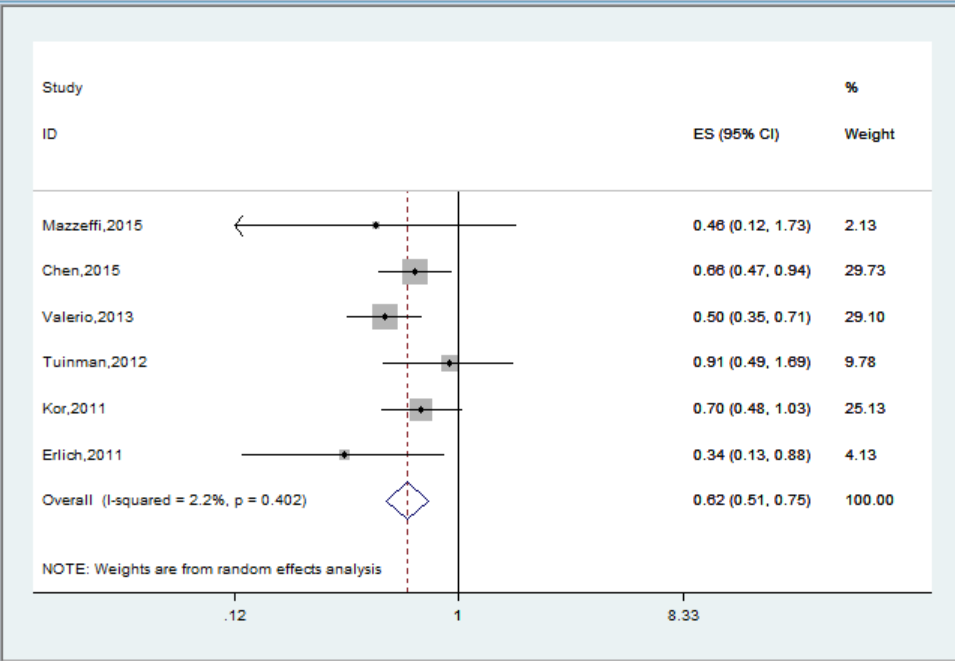


**Figure S19. The Sensitivity Analysis of the the Observational Studies**
